# Mortality and Clinical Outcomes of Aspergillus and COVID-19 Co-infection: A Retrospective Analysis

**DOI:** 10.7759/cureus.50321

**Published:** 2023-12-11

**Authors:** Juliana Cazzaniga, Nicole Koutras, Premalkumar Patel

**Affiliations:** 1 Medicine, Florida International University, Herbert Wertheim College of Medicine, Miami, USA; 2 Infectious Disease, Mount Sinai Medical Center, Miami, USA

**Keywords:** disseminated aspergillosis, invasive aspergillosis, pulmonary aspergillosis, covid-19, covid-19-associated pulmonary aspergillosis (capa)

## Abstract

Introduction: Patients with coronavirus disease (COVID-19) are more susceptible to secondary infections. Aspergillus co-infection has emerged as one of the most alarming complications in critically ill COVID-19 patients due to the disease itself or the use of immunomodulators and immunosuppressants for treatment. This study aimed to examine the mortality rates and clinical outcomes associated with Aspergillus and COVID-19 co-infection using data obtained from the largest nationwide inpatient sample database in the United States.

Methods: The National (Nationwide) Inpatient Sample (NIS) database is a vast and openly accessible collection of data that records millions of hospital admissions in the United States. For our research, we utilized the NIS 2020 database to identify adult patients diagnosed with COVID-19 and categorized them based on co-infection with Aspergillus. To examine the NIS database, we utilized various statistical methods such as univariate and multivariate analyses, descriptive statistics, and regression analysis.

Results: Of the 16.7 million patients hospitalized due to COVID-19 infection, 1485 developed Aspergillus co-infection. The demographics showed a predominance of males with 920 males and 565 women. A total of 46% were Caucasians, 17.2% were African-Americans, and 29.5% were Hispanics. The most common comorbidities were chronic pulmonary disease (40.7%), hypertension (41.4%), diabetes with chronic complications (37.7%), leukemia (4.0%), lymphoma (3.7%), and solid tumors (3.7%). Hospital mortality with co-infection was 53.2%, length of stay (LOS) 26.9 days, and economic utilization $138,093 in comparison to patients without co-infection with in-hospital mortality of 13.2%, LOS of 7.9 days, and cost of 21,490. Age-adjusted mortality was 6.6 (confidence interval: 5.9-7.3).

Conclusion: Our study indicated that the mortality rate in COVID-19 patients with Aspergillus infection was four-fold higher. Furthermore, comorbidities, such as diabetes mellitus, chronic pulmonary disease, and obesity, have been associated with worse outcomes. Further research is necessary to understand the etiological relationship between Aspergillus infection and COVID-19 in order to develop effective treatment strategies that mitigate the impact of this lethal combination on patient health outcomes.

## Introduction

The co-occurrence of COVID-19 and Aspergillus co-infections has drawn increasing attention in the medical community. Current understanding suggests that individuals with severe COVID-19, particularly those with compromised immune systems, are at an elevated risk of developing secondary infections, including those caused by the Aspergillus species [1]. Aspergillus, commonly found in the environment, can lead to invasive pulmonary aspergillosis (IPA) in susceptible individuals [1]. However, the precise mechanisms of interaction between COVID-19 and Aspergillus, as well as the factors influencing susceptibility, remain active areas of research. Addressing these knowledge gaps is crucial for enhancing clinical management strategies and improving outcomes for individuals facing the dual challenge of COVID-19 and Aspergillus co-infections.

Patients with COVID-19 are at increased risk of developing secondary bacterial, viral, and fungal infections [1]. COVID-19-associated pulmonary aspergillosis (CAPA) is a co-infection that has been reported to occur in up to 30% of hospitalized patients with critical COVID-19 infection [2]. COVID-19 patients and immunomodulators administered as part of the treatment have both been implicated as risk factors for developing CAPA [3]. The mortality rate for CAPA has been shown to be approximately 60%, with the rate being lower for those who received antifungals than for those who did not [4].

Associations between viral respiratory tract infections and aspergillosis have been previously demonstrated in influenza [4]. Influenza-associated pulmonary aspergillosis (IAPA) has an incidence of up to 30% and a mortality rate of approximately 50% [5]. However, patients with CAPA differ from those with classic IPA. Patients with IPA tend to have hematological malignancies or neutropenia. Those with CAPA, tend to be immunocompetent and have comorbidities, such as diabetes, hypertension, and heart disease [3].

The exact pathophysiology of CAPA is not known; however, it is thought that it more closely resembles the pathophysiology of IAPA than IPA [3]. In IAPA, there is a decreased ability of the lungs to effectively clear Aspergillus spp. This is partly, due to defective mucociliary clearance, reductions in nicotinamide-adenine dinucleotide phosphate (NADPH) oxidase, and dysfunction of the immune system due to local hypoxic factors. All these factors favor the invasion of Aspergillus spp [3]. COVID-19 damages the lungs in a manner similar to influenza to facilitate the growth of Aspergillus spp [3]. Another aspect of the pathophysiology of CAPA is the use of immunomodulators, such as tocilizumab, in the treatment of COVID-19. Tocilizumab is an anti-IL-6 receptor antibody used in COVID-19 pneumonia and acute respiratory distress syndrome (ARDS) [1].

Corticosteroids and IL-6 inhibitors, such as tocilizumab, contribute to the development of IPA through a variety of mechanisms [3,6,7]. Corticosteroids mainly interfere with phagolysosome maturation, and IL-6 inhibitors interfere with Th17 cell development [3]. During Aspergillus fumigatus infection, there is a substantial increase in IL-6 as a protective immune response. IL-6 inhibitors dampen the immune response to the fungus [8]. On the other hand, excessive IL-6 can have negative impacts on patients, as seen in ARDS. Therefore, IL-6 inhibitors have been used in patients with severe COVID-19, although they can increase the risk of developing pulmonary aspergillosis [8].

## Materials and methods

For this study, we utilized the NIS database as the primary data source. The NIS is a reliable and comprehensive database that provides data from approximately eight million hospital stays each year, making it the largest all-payer inpatient care database in the United States (US). It is derived from the billing data provided in the State Inpatient Databases and is stratified to be representative of approximately 20% of US community hospitals. We conducted a thorough analysis of the NIS database to investigate our research questions and obtain valuable insights.

For our research, we utilized the NIS 2020 database to identify adult patients diagnosed with COVID-19 and categorized them based on co-infection with Aspergillus. To examine the NIS database, we utilized various statistical methods such as univariate and multivariate analyses, descriptive statistics, and regression analysis.

The study utilized a retrospective cohort design, drawing data from the NIS 2020 database. This design involved the analysis of a pre-existing dataset to assess the association between COVID-19 and Aspergillus co-infection. The cohort comprised adult patients diagnosed with COVID-19, categorized based on the presence or absence of Aspergillus co-infection. This observational approach allowed for the examination of mortality rates and clinical outcomes associated with Aspergillus and COVID-19 co-infection in a large-scale, diverse patient population.

The study adopted a retrospective cohort design, utilizing data from the NIS 2020 database to examine the influence of Aspergillus co-infection on COVID-19 outcomes in adults. Inclusion criteria comprised adult patients aged 18 and above with a confirmed COVID-19 diagnosis, ensuring data completeness for essential variables. Pediatric cases were explicitly excluded, along with instances featuring incomplete records. The statistical analysis employed a multifaceted approach. Descriptive statistics were employed to provide an overview of demographic characteristics, comorbidities, and relevant factors in both the Aspergillus co-infection and non-co-infection groups. Univariate analyses compared demographic features, comorbidities, and outcomes between the two groups. Multivariate analysis was utilized to control for confounding variables, while regression analysis explored relationships and identified predictors of mortality and other crucial outcomes. This inclusive methodological framework facilitated a comprehensive exploration of the associations between COVID-19, Aspergillus co-infection, and diverse clinical outcomes within the studied population.

According to the NIS database, pre-defined comorbidity included in the Elixhauser Comorbidity Index is taken into account. The population with common immunocompromised conditions like liquid and solid malignancies, diabetes mellitus with and without complications, and chronic pulmonary diseases are outlined in the table. Our study does not exclude HIV, as Aspergillus and other fungal infections in HIV patients tend to be very indolent, and our study centers on Aspergillus infections during hospital admissions for severe COVID-19. The limitation of the database is that it does not differentiate between hospital-acquired and non-hospital-acquired conditions.

The main research question asked if there was an association between Aspergillus and COVID-19 co-infection and adverse outcomes compared with COVID-19 patients without Aspergillus co-infection.

Aspergillus co-infection in patients with COVID-19 was found to be associated with higher mortality rates, lengths of hospital stay, and healthcare costs compared to COVID-19 patients without Aspergillus co-infection. 

This fits within the existing literature that showed increased adverse outcomes in patients co-infected with COVID-19 and Aspergillus. It also fits with the pre-pandemic literature of worse outcomes for patients co-infected with influenza and Aspergillus. 

## Results

Analysis of the NIS database revealed several significant findings. The results of our analysis showed that among the 16.7 million patients hospitalized due to COVID-19 infection, 1,485 developed Aspergillus co-infection. The demographic analysis showed a predominance of males compared to females. Out of the 1,485 patients with Aspergillus co-infection, 920 were male and 565 were female. The racial distribution of among these patients with Aspergillus co-infection was 46% Caucasian, 17.2% African-American, and 29.5% Hispanic. Infection rates in the south (30.0%) and west (32.0%) of the United States were the highest when compared with those in the northeast (12.5%) and midwest (25.6%).

The most common comorbidities observed in these patients with Aspergillus co-infection were chronic pulmonary disease (40.7%), hypertension (41.4%), diabetes with chronic complications (37.7%), leukemia (4.0%), lymphoma (3.7%), and solid tumors (3.7% each). In-hospital mortality among patients with Aspergillus co-infection was found to be 53.2%, with a significantly longer length of stay (26.9 days) and higher economic utilization ($138,093) compared to patients without Aspergillus co-infection. The age-adjusted mortality among patients with Aspergillus co-infection was 6.6 (confidence interval 5.9-7.3). Table 1 presents data related to COVID-19 patients and their characteristics. It provides information on variables such as Aspergillus infection, age, gender, race, income quartile by zip code, insurance type, comorbidities, geographic region, hospital ownership/control, discharge quarter, and outcomes. Table 1 includes statistics, percentages, and Absolute Standardized Differences (ASD%) for various categories, facilitating a comprehensive analysis of the patient population.

**Table 1:** Demographics ASD: Absolute Standardized Differences

Variable	Aspergillus		No Aspergillus	ASD %
Total patients with COVID-19: 1678995	n = 1,485	n = 1677510		
Age				
Mean years (SE (standard error))	64.6±18.4	62.6±18.4		
Gender				
Male	920(61.9%)	872485(52%)		< .0001>
Female	565(38.1%)	804950(47.9%)		
Race				
Caucasians	655(46.0%)	825070(50.7%)		< .0001>
African-Americans	245(17.2%)	310535(19.1%)		
Hispanic	420(29.5%)	353025(21.7%)		
Income quartile by zip code				
0 – 25^th^	430(29.4%)	564095(34.2%)		< .0001>
26 – 50^th^	375(25.7%)	448625(27.2%)		
51 – 75^th^	380(26.0%)	365480(22.1%)		
76 – 100^th^	275(18.8%)	272380(16.5%)		
Insurance type				
Medicare	780(52.7%)	839665(50.1%)		< .0001>
Medicaid	280(18.9%)	252485(15.1%)		
Blue Cross PPO (preferred provider organization)	355(24.0%)	441060(26.3%)		
Elixhauser comorbidities				
Chronic pulmonary disease	605(40.7%)	360170(21.5%)		< .0001>
Hypertension	615(41.4%)	445600(26.6%)		< .0001>
Diabetes w/o chronic complications	80(5.4%)	225740(13.5%)		< .0001>
Diabetes w/ chronic complications	560(37.7%)	437120(26.1%)		< .0001>
Hypothyroidism	180(12.1%)	218940(13.1%)		< .0001>
Obesity	305 (20.5%)	439085(26.1%)		< .0001>
Cancer, leukemia	60(4.0%)	10760(0.6%)		< .0001>
Cancer, lymphoma	55(3.7%)	12780(0.8%)		< .0001>
Cancer, solid	55(3.7%)	29355(1.7%)		< .0001>
Depression	125(8.4%)	182375(10.9%)		< .0001>
Geographic region				
Northeast	185(12.5%)	308380(18.4%)		< .0001>
Midwest	380(25.6%)	373315(22.2%)		
South	445(30.0%)	689731(41.1%)		
West	475(32.0%)	306084(18.2%)		
Hospital ownership/control				
Rural	35(2.4%)	162981(9.7%)		< .0001>
Urban nonteaching	205(13.8%)	310095(18.5%)		
Urban teaching	1245(83.8%)	1204434(71.8%)		
Discharge quarter				
1^st^ Quarter	0	0		
2^nd^ Quarter	285(19.2%)	416410(24.9%)		
3^rd^ Quarter	410(27.6%)	427015(27.6%)		
4^th^ Quarter	790(53.2%)	831780(49.6%)		

Table 2 provides key insights into patient outcomes, including mortality rates, length of hospital stays, associated costs, and post-hospitalization dispositions. The significant p-values suggest statistically meaningful differences in these outcomes among the patient groups in Table 2. 

**Table 2:** COVID-19 outcomes and P-value

Outcomes	Aspergillus	No Aspergillus	P- Value
In-hospital mortality	780(52.5%)	221835(13.2%)	< .0001>
Length of stay, days	26.9±21.4	7.9±9.4	< .0001>
Total hospitalization cost, $	138,093±152,017.4	21,490±38,726.2	< .0001>
Disposition			
Discharge to home	165(11.1%)	863375(51.5%)	< .0001>
Transfer other: includes skilled nursing facility (SNF), intermediate care facility (ICF), and other types of facility	49(3.8%)	50455(3.0%)	
Discharged to home healthcare	315(21.2%)	307560(18.3%)	
Age-adjusted mortality	6.6 (CI 5.9 – 7.3 )		

**Figure 1:**
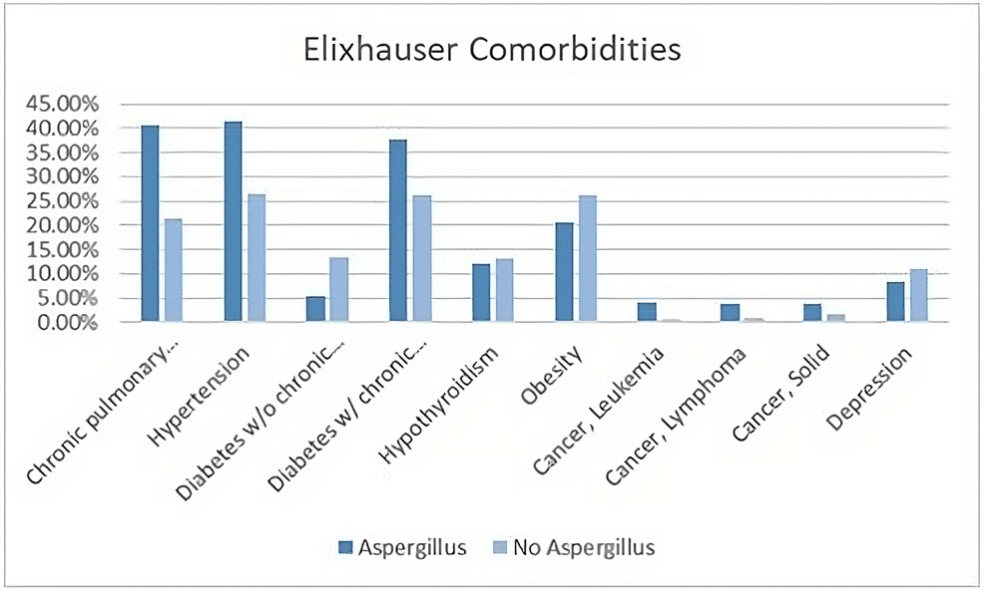
Elixhauser comorbidities in patients with and without Aspergillus infection Comorbidities: chronic obstructive pulmonary disease, hypertension, diabetes with chronic obstructive pulmonary disease, hypothyroidism, obesity, cancer (leukemia), cancer (lymphoma), cancer (solid), depression.

A comparison of Elixhauser comorbidities in patients with and without Aspergillus infection reveals that there is a similarity in the prevalence of various comorbidities as shown in Figure 1. Both groups, with and without Aspergillus, exhibited notable presence of chronic obstructive pulmonary disease (COPD), hypertension (HTN), diabetes, hypothyroidism, obesity, cancer, and depression. Such findings are valuable for healthcare professionals, as they suggest that when addressing these comorbidities in patients with Aspergillus, their management should not substantially differ from those without Aspergillus infection. However, individual patient assessments and tailored treatments should be considered to provide the best care and outcomes for each patient.

**Figure 2:**
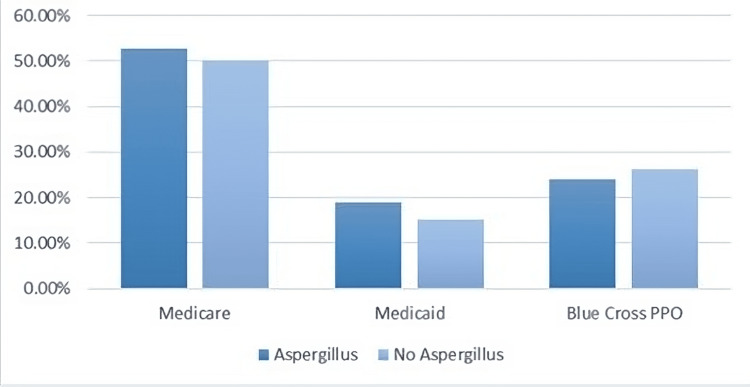
A comparison of insurance types among patients with and without Aspergillus infection

A comparison of insurance types among patients with and without Aspergillus infection reveals interesting insights as shown in Figure 2. Medicare, Medicaid, and Blue Cross insurance plans were common across both groups. This suggests that the presence or absence of Aspergillus infection does not significantly affect the distribution of insurance types. It is important to note that this finding underscores the importance of access to healthcare resources for individuals, regardless of their Aspergillus status. Additionally, it highlights the role of these insurance providers in supporting a broad spectrum of patients, including those facing complex medical conditions, such as Aspergillus infections. Individual patient cases may vary, and healthcare providers should continue to offer tailored care based on the specific needs and conditions of each patient, irrespective of their insurance type. 

**Figure 3:**
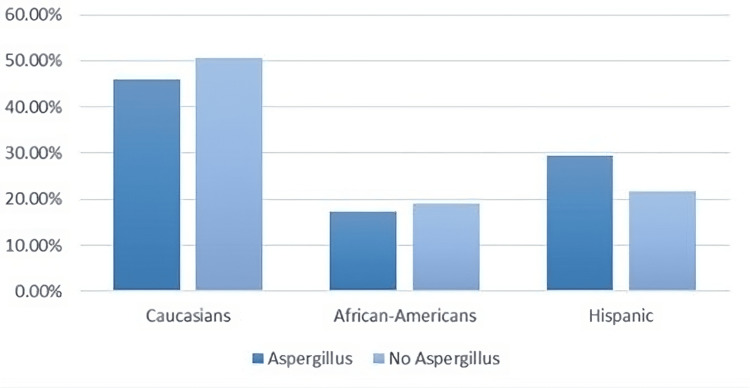
Examination of demographics among patients with Aspergillus infection

The examination of demographics among patients with Aspergillus infection and those without revealed interesting patterns in terms of race and ethnicity as shown in Figure 3. Among Caucasians, African Americans, and Hispanics, there appears to be a varied distribution in both populations. This finding demonstrates that Aspergillus infection does not disproportionately affect any specific racial or ethnic group. These findings underscore the importance of considering the diverse backgrounds of patients when addressing Aspergillus infection. It is crucial for healthcare providers to provide equitable care and consider the unique cultural and genetic factors that might play a role in patient outcomes. Furthermore, these findings reinforce the importance of promoting awareness and education about Aspergillus infections across all communities to ensure early detection and appropriate treatment, regardless of racial or ethnic background.

## Discussion

The study discussed here delves into the critical issue of co-infection between COVID-19 and Aspergillus, revealing its potential lethality and clinical implications [9,10]. As secondary infections in COVID-19 patients have become a growing concern, Aspergillus, a mold, has emerged as a worrisome complication [10]. The study's findings of this study emphasize the importance of this co-infection, with mortality rates ranging from 30% to 90% [10]. To conduct this research, the study utilized the extensive NIS database, known for aggregating millions of hospital admissions in the United States, employing various statistical methods for analysis [10]. The key findings of this study shed light on the demographics and comorbidities among patients with COVID-19 and Aspergillus co-infection [11]. Males were more susceptible, with 920 male patients compared to 565 female patients, and the racial distribution revealed a majority of Caucasians (46%), followed by Hispanics (29.5%), and African-Americans (17.2%) [11]. The study also noted prevalent comorbidities, with chronic pulmonary disease, hypertension, and diabetes, and chronic complications being the most common [11]. A smaller percentage of patients presented with leukemia, lymphoma, and solid tumors, indicating vulnerability among immunocompromised individuals [11].

This vulnerability among immunocompromised patients is important, given that immunocompromised states can be induced through the use of IL-6 inhibitors and corticosteroids. Both classes of medications are known to contribute to the development of pulmonary aspergillosis [3,6,7,8,9]. Corticosteroids impair the maturation of phagolysosomes involved in the degradation of Aspergillus fumigatus, weakening the immune system against the fungus [3]. Meanwhile, IL-6 inhibitors contribute to the development of Aspergillosis by inhibiting Th17 cell development, which is part of the immune response against Aspergillus fumigatus [3].

Both corticosteroids, such as dexamethasone, and IL-6 inhibitors are used to treat severe COVID-19 infections. Excessive amounts of inflammatory cytokines have negative effects on patient health [9]. They can induce ARDS and cardiac arrhythmias [8]. The goal of therapy with these two classes of medications was to reduce the amount of inflammatory cytokines to improve morbidity and mortality in patients with severe COVID-19 [9]. However, these medications are not without complications, one of which is pulmonary aspergillosis [8,9]. Thus, the benefits and risks must be weighed for each patient before starting these medications.

However, the most striking finding pertained to the mortality rates [12]. The in-hospital mortality rate among patients with Aspergillus co-infection was alarmingly high at 53.2%, in stark contrast to the 13.2% mortality rate observed in COVID-19 patients without Aspergillus co-infection [12]. These statistics underscore the life-threatening nature of this co-infection [12]. Additionally, the study examined economic implications, revealing longer hospital stays (26.9 days) and significantly higher costs ($138,093) for patients with Aspergillus co-infection [12].

Age-adjusted mortality, reported at 6.6 with a confidence interval of 5.9 to 7.3, suggests that age plays a role in the severity of outcomes in these patients [12]. In conclusion, this study emphasizes the urgent need to address Aspergillus co-infection in COVID-19 patients and highlights the four-fold increase in mortality rates compared to those without this co-infection [12]. It also underscores the importance of managing comorbidities, such as diabetes, chronic pulmonary disease, and obesity, which are associated with worse outcomes [12]. The study's implications extend to clinical practice and research, emphasizing the need for comprehensive patient care and further investigations to better understand the interaction between Aspergillus infection and COVID-19, ultimately leading to more effective treatment strategies [12]. Comorbidities, including cardiovascular diseases and diabetes, amplify the risk of severe complications in COVID-19 patients, while advanced age, particularly beyond 65, accentuates vulnerability to severe manifestations such as pneumonia and acute respiratory distress syndrome. 

In the realm of our research focus, it is crucial for future studies to delve into a comprehensive characterization of the lung microbial ecosystem [13]. It is essential to conduct more extensive comparative studies to assess the influence of CAPA on mortality, specifically in ventilated patients [14]. Subsequent research designs should incorporate strategies focused on creating enhanced diagnostics for assessing tissue invasion and airway involvement [15]. 

Several limitations should be acknowledged in interpreting the findings of this study. Firstly, the retrospective nature of the analysis using the NIS 2020 database inherently relies on the accuracy and completeness of the recorded data, which might be subject to variations in coding practices across different healthcare facilities. Future research can consider using prospective study designs that directly capture detailed clinical information. This would minimize variations in the retrospective nature and potential data inaccuracies associated with databases. Another idea would be to include a more inclusive sampling approach, encompassing both hospitalized and outpatient cases. This would mitigate selection bias and provide a comprehensive understanding of Aspergillus co-infection's impact across diverse disease severities. Additionally, longitudinal studies could explore causal relationships, elucidating dynamic interactions between COVID-19, Aspergillus co-infections, and outcomes. Another future study could include pediatric cases and documenting specific antifungal treatments would improve generalizability and offer insights into treatment modalities.

The inclusion of only hospitalized patients may introduce a selection bias, excluding milder cases managed on an outpatient basis. Moreover, the study design does not allow for establishing causal relationships, and the observed associations should be interpreted as correlational. The exclusion of pediatric cases might limit the generalizability of the findings to the entire population. Finally, the study does not delve into the specific antifungal treatments administered, which could impact outcomes. Despite these limitations, the study contributes valuable insights into the association between Aspergillus co-infection and COVID-19 outcomes, prompting the need for further prospective research to validate and expand upon these findings.

## Conclusions

The presence of Aspergillus co-infection in COVID-19 patients was associated with higher mortality rates, longer hospital stays, and increased healthcare costs compared to patients without co-infection. These results emphasize the need for close monitoring and early detection of Aspergillus co-infection in COVID-19 patients, as well as the importance of implementing measures to prevent and manage this complication to improve patient outcomes and reduce the burden on healthcare resources. Further research is required to determine the role of steroids and IL-6 inhibitors in the increasing incidence of Aspergillus infections.
